# Inhibitory Effect of FMRFamide on NO Production During Immune Defense in *Sepiella japonica*

**DOI:** 10.3389/fimmu.2022.825634

**Published:** 2022-04-28

**Authors:** Libing Zheng, Huimin Cao, Jiayin Qiu, Changfeng Chi

**Affiliations:** National and Provincial Joint Laboratory of Exploration and Utilization of Marine Aquatic Genetic Resources, School of Marine Science and Technology, Zhejiang Ocean University, Zhoushan, China

**Keywords:** *Sepiella japonica*, FMRFamide, nitric oxide, inhibitory effect, immune response

## Abstract

Neuropeptide Phe-Met-Arg-Phe-NH_2_ (FMRFamide), specifically existing in invertebrates, plays pivotal roles in various physiological processes. The involvement in neuroendocrine-immune regulation was explored in recent years, and it could modulate nitric oxide (NO) production under immune stress. However, detailed knowledge is still little known. In this study, we identified FMRFamide as an inhibitory factor on NO production in the immune reaction of *Sepiella japonica*. Firstly, *Vibrio harveyi* incubation caused significantly upregulated expression of FMRFamide precursor and NO synthase (NOS) in just hatched cuttlefish with quantitative Real-time PCR (qRT-PCR), which indicated that both were likely to be involved in the immune defense. The whole-mount *in situ* hybridization (ISH) detected FMRFamide precursor and NOS-positive signals appeared colocalization, suggesting that at histological and anatomical levels FMRFamide might interact with NOS. Next, NOS mRNA was highly significantly upregulated at 72 h when FMRFamide precursor mRNA was knocked down effectively with the RNA interference (RNAi) method; the results hinted that FMRFamide was likely to regulate NO production. Continuously, the inflammatory model was constructed in RAW 264.7 cells induced by lipopolysaccharide (LPS), FMRFamide administration resulted in a highly significant reduction of the NO level in dose- and time-response manners. Although the addition of the selected inducible NOS (iNOS) inhibitor had inhibited the NO production induced by LPS, the additional FMRFamide could still furtherly sharpen the process. Collectively, it was concluded that neuropeptide FMRFamide could indeed inhibit NO production to serve as feedback regulation at the late stage of immune response to protect hosts from excessive immune cytotoxicity. The inhibitory effect on NO production could not only be mediated by the NOS pathway but also be implemented through other pathways that needed to be furtherly explored. The results will provide data for comparing the structure and immune function of neuroendocrine-immune system (NEIS) between “advanced” cephalopods and other invertebrates and will provide new information for understanding the NEIS of cephalopods.

## 1 Introduction

Phe-Met-Arg-Phe-NH_2_ (FMRFamide), specifically existing in invertebrates (1, 2), was firstly discovered from ganglion of *Macrocallista nimbosa* in 1977 (3). The common model of FMRFamide precursor contains a unique furin-processing site (RXK/RR) that typically separates the precursor into N- and C-terminal regions; N-terminus encodes a signal peptide, FL/IRFamide peptides, and a decapeptide; C-terminus harboring FMRFamides can be processed into mature peptides by proteases and then released to function in various physical processes (4). The studies of FMRFamide in cephalopods were started in 1987, Martin and Voigt (5) firstly identified FMRFamide in the venous serum of *Octopus vulgaris* central nervous system (CNS). In 1994, a report indicated that FMRFamide abundantly existed in the optic lobe of *Loligo chinensis* (6). Cosmo and Cristo (7) found that FMRFamide-positive signals existed in the CNS and peripheral nervous system (PNS) of *O. vulgaris*. In subsequent studies, it was found that invertebrate hemocytes also expressed and synthesize FMRFamide (7, 8) and some of its receptors (9). Therefore, the nervous system and some immune cells are the main sites to synthesize FMRFamide.

All the identified invertebrate nitric oxide synthases (NOSs) belong to one isoform sharing higher similarities to vertebrate neuronal NOS (nNOS), but it had resembled broad involvements in myriad physiological roles (10). An early study showed FMRFamide-positive nerve fibers connected to target cells through a non-synaptic form (11). Röszer et al. (12) observed that NOS-positive cerebral ganglia cells were stained by FMRFamide antibody; NOS-stained neurons also received FMRFamide immunoreactive terminals on soma and axonal processes in *Helix lucorum*, speculating that FMRFamide is likely to control nitric oxide (NO) generation at the histological and anatomical levels. Continuously, *in vitro* assay of the central ganglia showed FMRFamide administration caused a significant improvement in NO production, while NO content was reduced using FMRFamide-reactive IgGs to remove FMRFamide, and a similar result was obtained by inhibitor *N*^ω^-nitro-l-arginine (NOARG), or sodium channel inhibitor amiloride hydrochloride (AH) administration (13, 14). These data furtherly provided proof that in the nervous system of *H. lucorum*, FMRFamide could really regulate NO production through NOS, and the mediating effect was reached by activating its corresponding receptor to transduce the signals into cells.

As a key gaseous signal molecule, NO not only plays an important regulatory role in neuroscience but also mediates the immune response (15–17). NO can react indirectly with reactive oxygen species (ROS) to generate a much more powerful oxidant peroxynitrite (ONOO^−^) (18). NO has been considered to alter the redox status of hemolymph and increase oxidative toxicity by stimulation with cytokines, microbes, or bacterial products (10). In mollusks, the hemocyte phagocytosis was reported to be activated before NO-involved humoral defense (19). Norepinephrine (NE) could lower NO levels in the hemolymph of *Chlamys farreri* through activating its α receptor at the early immune response to lipopolysaccharide (LPS), and NO might regulate NE synthesis by a negative feedback pathway (20). NO content in the hemolymph of *Crassostrea gigas* continued to decline within 12 h post-injection FMRFamide (8). So No is not only involved in immune response directly but also plays crucial neuroendocrine-immune regulatory roles. FMRFamide could also regulate NOS activity, and intact neurons or sustained neural activity was not necessary for NO production mediated by FMRFamide (13). Hemocytes could synthesize FMRFamide and its receptors, so FMRFamide might also regulate NO production in the immune system.

*Sepiella japonica* has high edible, medicinal, and ecological values and used to be one of the four major kinds of seafood in the East China Sea. Artificial breeding has been carried out to recover its resources, but the mantle skin injuries in the aquaculture facilities are an advantage for the consequent development of infections during the artificial culture process, especially the skin canker disease caused by *Vibrio harveyi* (21). The neuroendocrine-immune regulatory study of FMRFamide in *S. japonica* will contribute to understanding its immune regulatory roles in cephalopods under stress. In the paper, based on mediating effect of FMRFamide on NO production, our aims were to 1) detect the induction expression of FMRFamide precursor and NOS postinfection by *V. harveyi*, 2) examine the colocalization of FMRFamide precursor with NOS in *S. japonica*, 3) perform RNA interference (RNAi) of FMRFamide precursor and analyze NOS expression change, and 4) explore NO-derived nitrite formation induced by FMRFamide in LPS-induced RAW 264.7 cells. The results will enrich the knowledge of mollusk neuroendocrine-immune system (NEIS) and provide data for comparing the structure and immune function of NEIS between “advanced” cephalopods and other invertebrates.

## 2 Materials and Methods

### 2.1 Animals

Healthy *S. japonica* adults were bought from a local cuttlefish breeding base (Xixuan Island, Zhoushan, Zhejiang Province) and temporarily reared in tanks containing aerated seawater. The cuttlefishes were fed twice with shrimp each day.

The juvenile cuttlefishes hatched about 30 days were anesthetized on ice. Optic lobes were dissected and fixed in 4% paraformaldehyde (PFA) immediately at 4°C overnight. The next day, optic lobes were dehydrated in gradient methanol in PTW (0.1% Tween 20 in 1× phosphate-buffered saline (PBS)) and then stored in methanol at −20°C for *in situ* hybridization (ISH) within 2 weeks.

Some ready-to-hatch eggs were transported to the lab and continued to be cultured in aerated seawater at 23°C ± 0.5°C. After 3–5 days, the cuttlefish larvae hatched and fed with *Brachiopoda rotifers*.

### 2.2 Challenge Experiment

In the challenge experiment, 40 cuttlefish larvae hatched within 3 days were incubated in 200 ml of aerated fresh seawater containing 2 × 10^7^ *V. harveyi* cells in the logarithmic growth phase. Nine individuals were randomly selected; every three were sampled together at 0, 3, 6, 12, 24, 48, and 72 h posttreatment and stored in Trizol at −80°C for RNA isolation.

### 2.3 Quantitative Real-Time PCR

#### 2.3.1 RNA Isolation and cDNA Synthesis

The challenged cuttlefish larvae at various times were ground, and total RNAs were isolated with TransZol Up (TransGen, Beijing, China). RNAs were transcribed to be cDNAs using PrimerScript™ RT reagent kit with gDNA Eraser (TaKaRa, Maebashi, Japan) according to the manufacturer’s instruction.

#### 2.3.2 Quantitative Real-Time PCR

According to the cDNA sequence of NOS obtained in the previous study (unpublished), a pair of primers was designed (**Table 1**). qRT-PCR using TB Green™ Premix Ex Taq™ II (Tli RNaseH Plus) kit on a Bio-Rad CFX connect system was run to explore the expression of FMRFamide precursor (22) and NOS postinfection by *V. harveyi*. Melting curve analysis after qRT-PCR confirmed the specificity of the primers. Both *β-actin* (23) and *EF-1γ* (24) were used as double internal controls. Three biological replicates were conducted, and each was carried out in triplicates. The relative expression level was calculated with the formula 2^−ΔΔCT^ (25). All data were given in terms of relative mRNA expression as mean ± SD (n = 3). The average fold-change in the mRNA level was calculated relative to the blank group. The statistical analysis was performed with a t-test or two-way ANOVA by IBM SPSS Statistics 21: the analysis yields three distinct *p*-values: i) whether the certain treatment had an effect, ii) whether the time had an effect, and iii) whether the effect of treatment changed with time.

**Table 1:** Primers used in the study.

Primers	Sequences (5′–3′)
**RT-PCR**	
*qSj-FMRF-F*	CGTCATCGCCATCTACTGTC
*qSj-FMRF*-R	CGCTTGCTTCTCAGTCCATC
*qSj-NOS*-F	GATAGTGCCACCAATCAGCG
*qSj-NOS*-R	CCTTGCCCATCAGTTTAGCC
*Sj-β-actin*-F	GCCAGTTGCTCGTTACAG
*Sj-β-actin*-R	GCCAACAATAGATGGGAAT
*Sj-EF-γ*-F	GCAAAAACCCCACCAAAGCCGA
*Sj-EF-γ*-R	AAGTGTTCCCAAAAGTACGGCA
**ISH**	
*ISj-FMRF*-F	CCCAAGCGTGATGCGTTGTTGGAGT
*ISj-FMRF*-R	CCGGAAGCGCTTGCTTCTCAGTCCATC
*ISj-NOS*-F	CAAGAAGCAACAGGAGC
*ISj-NOS*-R	AGGTAACGGGTGAAGGC
**RNAi**	
SiRNA-NC	AUUCGUUAGCUCGUGCACGTT
	CGUGCACGAGCUAACGAAUTT
SiRNA-FMRFamide	GCGAAGAGAAGAGGUUUAUTT
	AUAAACCUCUUCUCUUCGCTT

ISH, in situ hybridization; RNAi, RNA interference.

### 2.4 Colocalization Detection of FMRFamide Precursor and Nitric Oxide Synthase With Whole-Mount *In Situ* Hybridization

#### 2.4.1 Two Types of RNA Probes Preparation

According to the cDNA sequence of NOS, a pair of primers was designed (**Table 1**); the primers of FMRFamide precursor were employed in the previous study (22). The gel-purified PCR products were ligated into pGEM-T easy vectors (Invitrogen, Carlsbad, CA, USA), and the ligated products were subcloned into *Escherichia coli* DH5α. Positive clones were sequenced to ensure fragment correctness.

Next, the probes were synthesized with NTP-labeled uridine triphosphate (UTP) incorporated into the antisense RNA probe. The NTP labeling mix contains either DIG-11-UTPs (Roche, Basel, Switzerland) for the synthesis of DIG-labeled NOS probes or fluorescein-12-UTPs (Roche) for the synthesis of fluorescein-labeled FMRFamide probes. The fragments were amplified with sequenced plasmids as templates. The gel-purified products were employed to perform *in vitro* transcription in a 20-μl volume including 5× Transcription Buffer 2.0 μl, 10× Dig/Fluorescein RNA labeling Mix 2.0 μl, PCR products 1.0 μg, RNase Inhibitor (40 U/μl) 1.0 μl, T7 RNA polymerase 2 μl, and diethyl pyrocarbonate (DEPC) H_2_O up to 20 μl. The reactions were kept at 37°C for 2.5 h; then 2.0 μl of DNase I was added to keep another 30 min. The concentration and integrity of purified RNA probers were checked by spectrophotometer (A260/A280) and 1.2% agarose gel electrophoresis, respectively. They were stored at −80°C until use.

#### 2.4.2 Sample Processing and Probe Hybridization

All procedures in the report of Jezzini et al. (26) were employed with some modifications. All were performed at room temperature except as otherwise noted.

On the first day, dehydrated optic lobes were rinsed, digested in protease K for about 2 h at 37°C, and post-fixed in 4% PFA. Glycine buffer (2 mg/ml in PTW) was used to stop the above reactions. After being serially bathed in TEA–HCl (0.1 M of triethanolamine hydrochloride, pH 8.0) and TEA and acetic anhydride mixture, samples were treated in pre-hybridization buffer (50% formamide, 5 mM of ethylenediaminetetraacetic acid (EDTA), 5× SSC, 1× Denhardt’s solution, 0.1% Tween-20, and 0.5 mg/ml of yeast tRNA) at 50°C for 6–8 h, and then fresh pre-hybridization buffer containing about 3 ng/μl of RNA probes (denatured at 80°C for 5 min) was prepared to perform hybridization for 12–14 h at 50°C.

On the second day, after serial rinsing, samples were blocked in polybutylene terephthalate (PBT) (0.1% Triton-X 100, 2 mg/ml of bovine serum albumin in PBS, pH 7.4) for 3 × 20 min/time and PBT containing 10% goat serum for 90 min at 4°C. Then samples were incubated with anti-fluorescein-AP Fab antibody in PBT containing 1% goat serum (1:1,000) for 12–14 h at 4°C.

On the third day, all samples were rinsed in filtered Fast Red detection buffer (FRDB: 0.1% Tween 20, 100 mM of Tris–HCl; pH 8.2) for 3 × 5 min/time at 4°C and transferred to FRDB containing substrate Fast Red (Fast Red TR/naphthol AS-MX, Sigma, St. Louis, MO, USA). Colorimetry development (red) was monitored closely and terminated in PBT, and images were taken. Next, the residual alkaline phosphatase activity was inactivated in 0.1 M of glycine hydrochloride for 10 min. Anti-Dig-AP Fab antibody in PBT containing 1% goat serum (1:1,500) was added to incubate for another 12–14 h at 4°C.

On the fourth day, the chromogenic procedures with NBT/BCIP (purple) were performed as in a previous report (27). The signals were imaged under a stereomicroscope (Leica, Wetzlar, Germany).

### 2.5 RNA Interference

The siRNA-FMRFamide or its control siRNA (siRNA-NC) was designed and synthesized (GenePharma, Shanghai, China); the corresponding primers are shown in **Table 1**. The synthesized siRNAs were dissolved in 20 μM of sterile 0.9% NaCl right before they were used.

In the experiment, healthy cuttlefishes (weight 61.9 ± 13.8 g, mantle length 8.2 ± 0.7 cm) were divided into four groups. The first was a blank group with no treatment; the second is a saline group that received an injection with 50 μl 0.9% of NaCl; the third is an RNAi group that received 50 μl of a solution containing 30 μl 0.9% of NaCl, 10 μl of Lipo6000™, and 10 μl of siRNA-FMRFamide (20 μM); and the fourth group comprised cuttlefishes injected with the same concentration of siRNA-NC. Cuttlefishes were anesthetized in MgCl_2_ (17 g/L) for about 30 s before injection, and the injection site was in the main vein (28). The hemolymph was collected with a sterile syringe containing marine anticoagulation (18) from the main vein at 0, 24, 48, and 72 h postinfection and centrifuged at 800 g for 10 min at 4°C to obtain hemocytes for total RNA isolation and cDNA synthesis.

### 2.6 Colorimetric Detection Effect of FMRFamide on Nitric Oxide in Lipopolysaccharide-Induced RAW 264.7 Cells

#### 2.6.1 FMRFamide Administration in Lipopolysaccharide-Induced RAW 264.7 Cells

RAW 264.7 cells were maintained in Dulbecco’s modified Eagle medium (DMEM) supplemented with 10% fetal bovine serum (FBS) in a 5% CO_2_ atmosphere at 37°C. The cells were passaged onto a 96-well cell culture plate and adherently cultured for 24 h. Next, 10^−3^–10 μM (final concentration) of chemically synthesized FMRFamide (Sangon Biotech, Shanghai, China) was added into wells to pretreat for 15 min; 20 μl of LPS was added to a final concentration of 1 μg/ml and cultured for another 24 h. Or RAW 264.7 cells were incubated with 0.01 μM of FMRFamide and LPS for 3, 6, 12, 24, 48, and 72 h. Wells with no FMRFamide were used as controls. Each group was set at three repeats. The cell supernatant was collected into a sterile 1.5-ml centrifuge tube, and NO was detected using NO assay kit (Nanjing Jiancheng, Nanjing, China) by the Griess reaction to form a light-red azo compound.

In addition, 10 μM of NOS inhibitors of *S*-methylisothiourea sulfate (SMT; selective inhibitor of inducible NOS (iNOS); Beyotime, Shanghai, China) was used to preincubate cells for 30 min; then LPS and FMRFamide were added to perform as described above. Three independent trials were conducted, and data were analyzed with one- or two-way ANOVA, followed by Dunnett’s multiple-comparisons test. The NO level was shown as mean ± SEM (n ≥ 3).

## 3 Results

### 3.1 The Induction Expression of FMRFamide Precursor and Nitric Oxide Synthase by *Vibrio harveyi*

**Figure 1:**
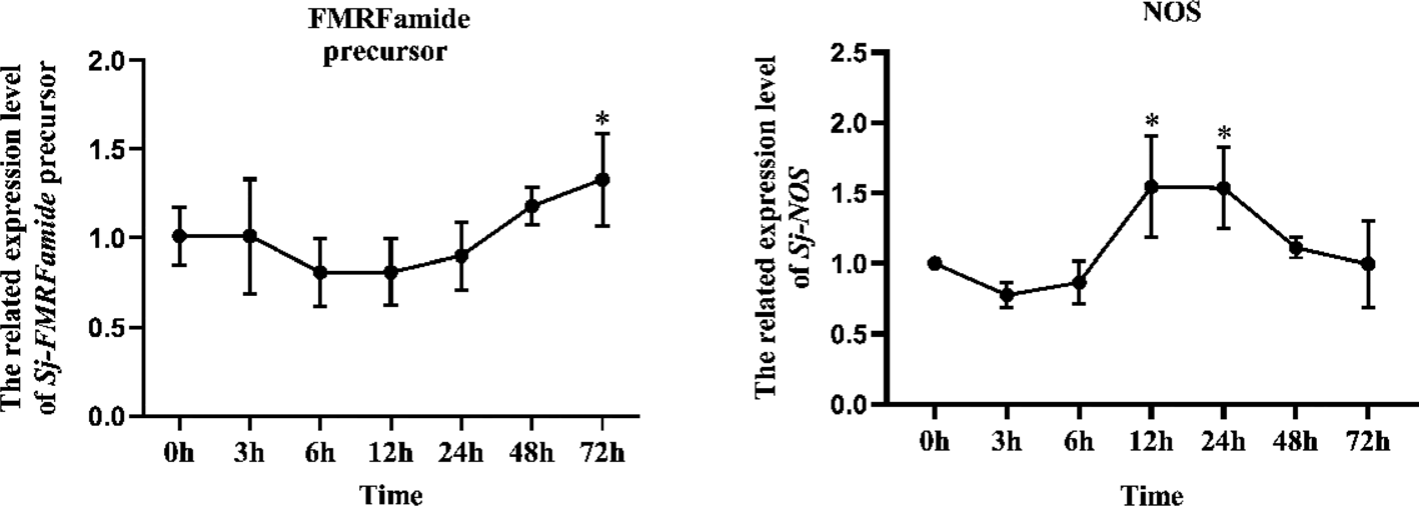
Induction expression of FMRFamide precursor and NOS in cuttlefish larvae induced by *Vibrio harveyi*. Data were analyzed by a t-test that compared the treatment group at any given time to the blank group. One asterisk indicates a significant difference (*p* < 0.05). NOS, nitric oxide synthase.

After being incubated with *V. harveyi*, the induced expression of FMRFamide precursor and NOS in cuttlefish larvae was analyzed; the results are shown in **Figure 1**. Compared to the blank group (0 h), after being stimulated by *V. harveyi*, the expression level of FMRFamide precursor was significantly upregulated to 1.32-fold (*p* < 0.05) till 72 h; NOS was upregulated at 12 and 24 h to 1.55-fold (*p* < 0.05) and 1.53-fold (*p* < 0.05), respectively, and returned to the initial level at 48 h. Collectively, the data showed that both FMRFamide precursor and NOS could be involved in the defense response against pathogen infection in hatched cuttlefish larvae.

### 3.2 Whole-Mount Colocalization of FMRFamide Precursor and Nitric Oxide Synthase

**Figure 2:**
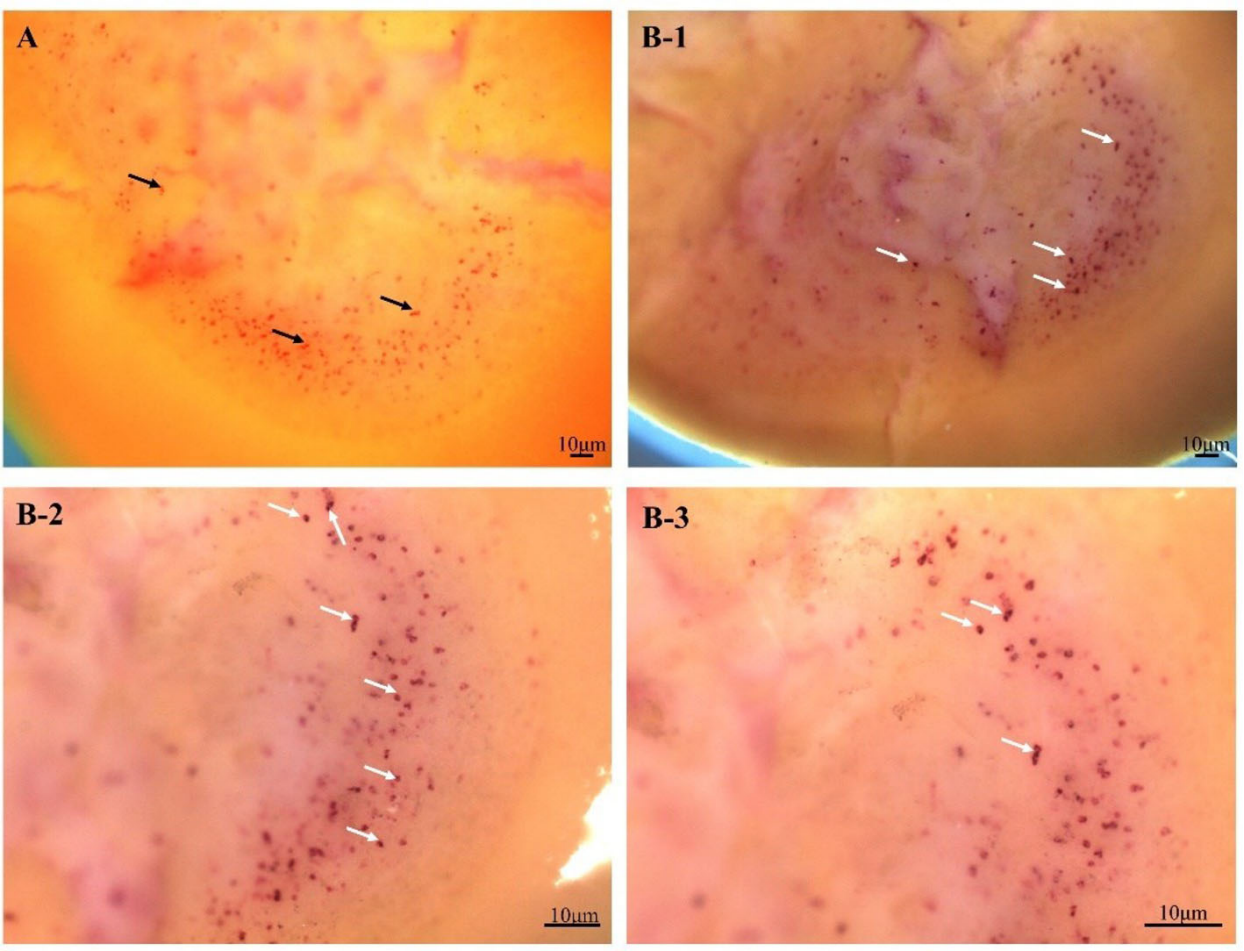
Colocalization result of FMRFamide precursor and NOS in the optic lobe. **(A)** FMRFamide precursor localization on the optic lobe. **(B)** Colocalization of FMRFamide precursor (red) and NOS (purple) on the optic lobe. **(A, B-1)** The ventral area of the optic lobe connecting to the brain. **(B-2/3)** The dorsal area of the optic lobe connecting to the retina. The black arrows show some representative FMRFamide precursor positive signals; the white arrows indicate some representative colocalization sites of FMRFamide precursor and NOS. NOS, nitric oxide synthase.

The colocalization of FMRFamide precursor (red) and NOS (purple) was detected with whole-mount ISH. Obviously, **Figure 2–A** shows the optic lobe had a lot of red positive signals stained with Fast Red indicating FMRFamide precursor, and the signals are mainly distributed in the medulla (A). Surprisingly, after chromogenic reaction with NBT/BCIP again, many purple signals indicating NOS emerged on red signals, suggesting that the two colors overlapped (**Figure 2–B–1/2/3**). The colocalization result implied that the FMRFamide is likely to interact with NOS.

### 3.3 FMRFamide Precursor Expression Post-RNA Interference

**Figure 3:**
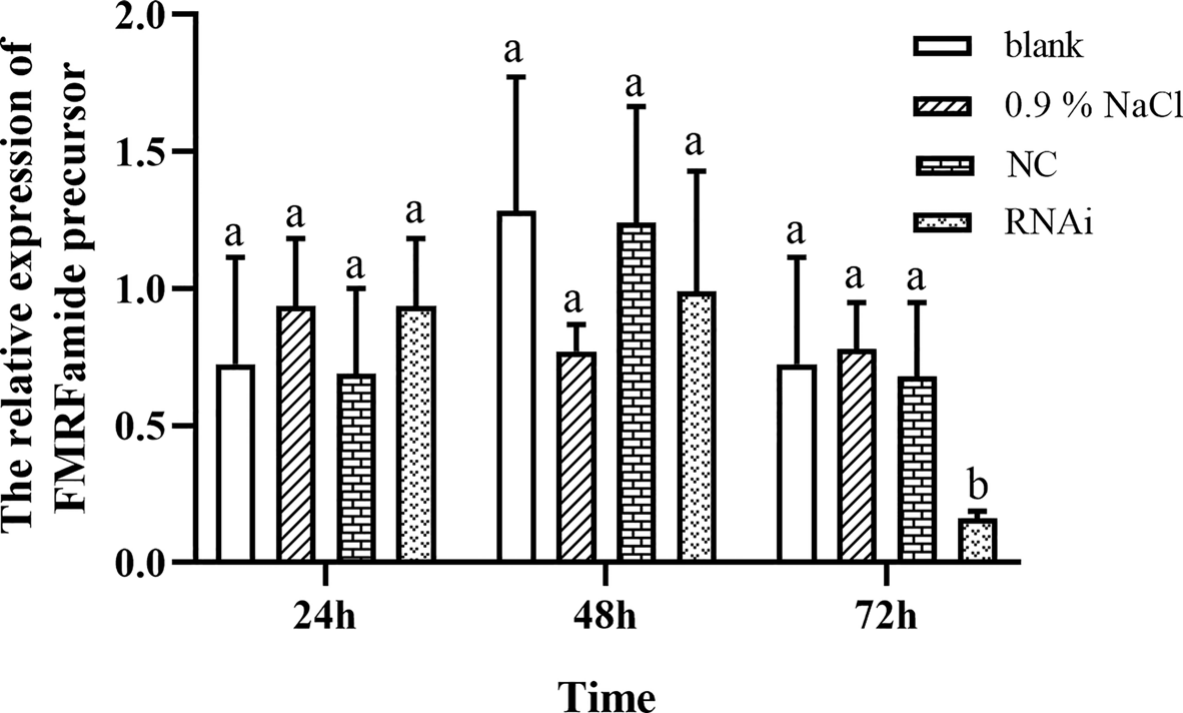
FMRFamide precursor expression change in hemocytes after RNAi precursor. Data were analyzed by two-way ANOVA followed by Dunnett’s *post-hoc* test. The same letter indicates no significant difference (*p* > 0.05); different letters indicate a significant difference (*p* < 0.05). RNAi, RNA interference.

RNAi experiment was carried out by injection siRNA-FMRFamide to knock down the FMRFamide precursor; the result is shown in **Figure 3**. Compared to control groups, 4 μM of siRNA-FMRFamide treatment resulted in significant downregulation of FMRFamide precursor at 72 h in hemocytes. Therefore, the data indicate that we have performed RNAi experiments successfully in this study, and the knockdown efficiency of FMRFamide precursor mRNA could be reached about 84%.

### 3.4 Nitric Oxide Synthase Expression Post-RNA Interference FMRFamide Precursor

**Figure 4:**
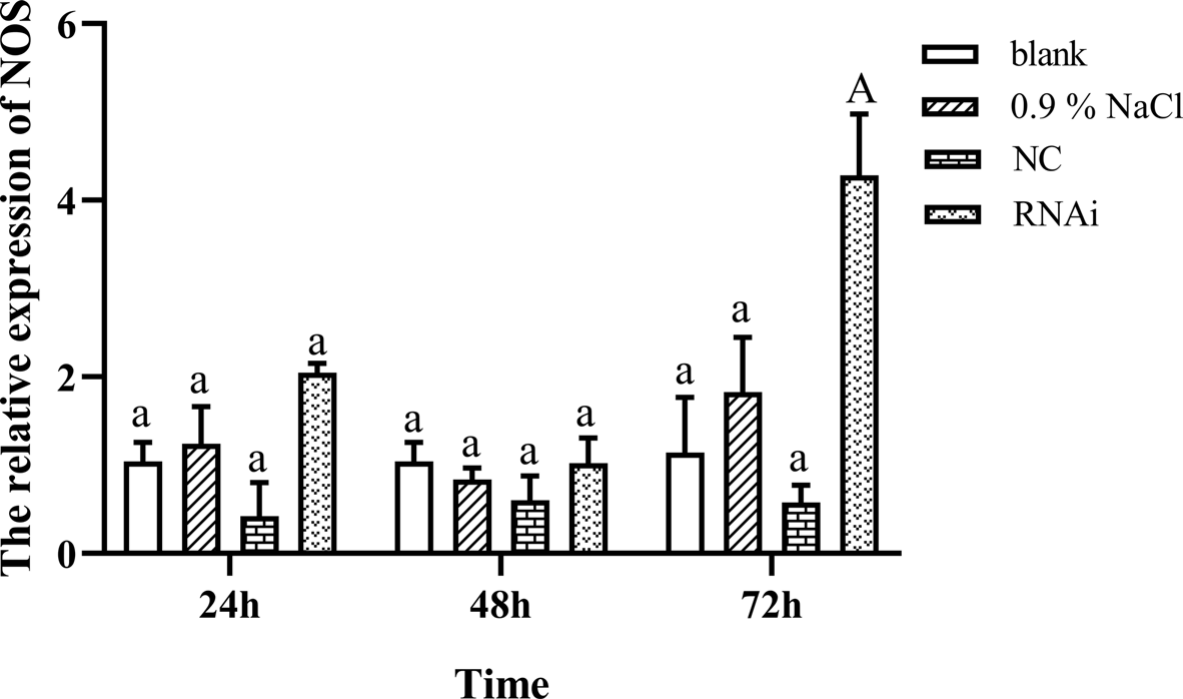
NOS expression after knocking down FMRFamide precursor mRNA. Data were analyzed by two-way ANOVA followed by Dunnett’s *post-hoc* test. The capital letter indicates a highly significant difference (*p* < 0.01); the same small letter indicates no significant difference (*p* > 0.05). NOS, nitric oxide synthase.

**Figure 4** shows the expression change of NOS after successfully knocking down FMRFamide precursor mRNA *in vivo*; surprisingly, the NOS expression level was highly significantly upregulated at 72 h when FMRFamide precursor mRNAs were knocked down effectively, which illustrated that FMRFamide precursor could inhibit NOS expression in *S. japonica*.

### 3.5 Inhibitory Effect of FMRFamide on Nitric Oxide Production in Inflammatory RAW 264.7 Cells

#### 3.5.1 Concentration-Dependent Inhibitory Effect

**Figure 5:**
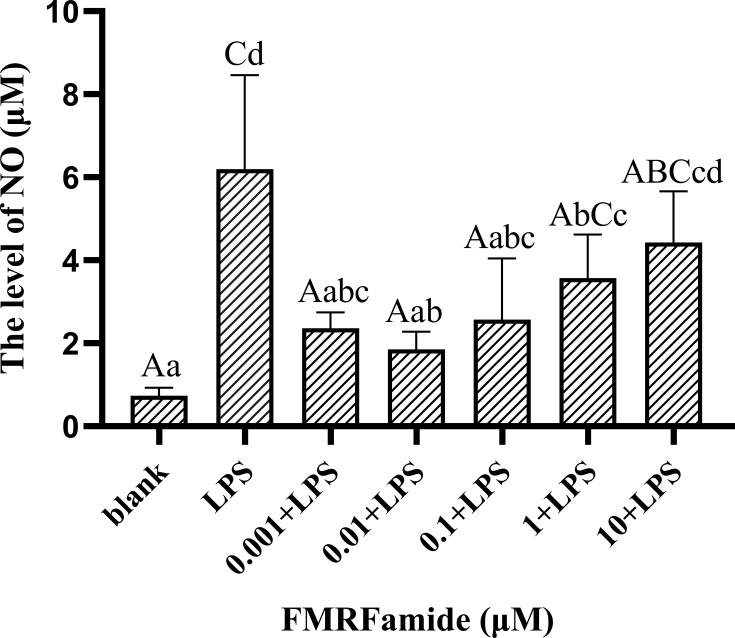
The effect of FMRFamide on NO production in inflammatory RAW 264.7 cells. The graph displays the mean ± SEM (n ≥ 3), and data were analyzed by one-way ANOVA followed by Dunnett’s *post-hoc* test. The different capital letters indicate highly significant difference (*p* < 0.01); different small letters indicate significant difference (*p* < 0.05); the same letter indicates no significant difference (*p* > 0.05). NO, nitric oxide.

The inflammatory model of RAW 264.7 cells induced by LPS was constructed successfully in this study to detect the effect of FMRFamide on NO production *in vitro*. Firstly, the treatment results of 10^−3^–10 μM of FMRFamide on the NO level are shown in **Figure 5**. Compared to the blank group, the NO level was highly significantly elevated post-LPS incubation alone, indicating that the inflammatory induction was effective. In turn, compared to the LPS groups, the NO level was highly significantly reduced after adding FMRFamide except in the 10 μM groups where it appeared to have a declining trend. The data clarified that FMRFamide could inhibit NO production under immune stress, and the inhibitory effect appeared to have a dose-dependent response within a certain range. The optimal induction concentration was 0.01 μM.

#### 3.5.2 Time-Dependent Inhibitory Effect

**Figure 6:**
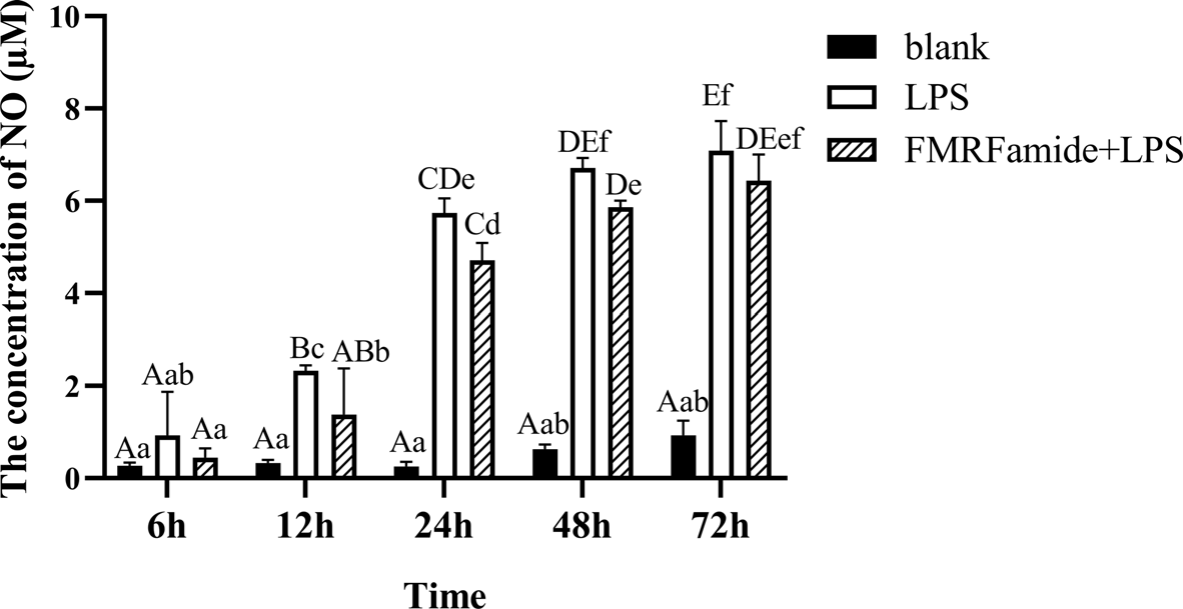
NO level induced by 0.01 μM of FMRFamide for different times in inflammatory RAW 264.7 cells. The graphs display the mean ± SEM (n ≥ 3). Data were analyzed by two-way ANOVA followed by Dunnett’s *post-hoc* test. The different capital letters indicate a highly significant difference (*p* < 0.01); different small letters indicate a significant difference (*p* < 0.05); the same letter indicates no significant difference (*p* > 0.05). NO, nitric oxide.

As mentioned above, inflammatory RAW 264.7 cells were treated with 0.01 μM of FMRFamide at different times in this experiment. As seen in **Figure 6**, the LPS groups induced highly significant NO content elevation with increased time duration, whereas the addition of 0.01 μM of FMRFamide significantly cut down NO production within 48 h, which implied that the inhibitory effect of FMRFamide on NO production under immune stress was also time-dependent.

### 3.6 Co-Stimulation Effect of *S*-Methylisothiourea Sulfate and FMRFamide on Nitric Oxide Level

**Figure 7:**
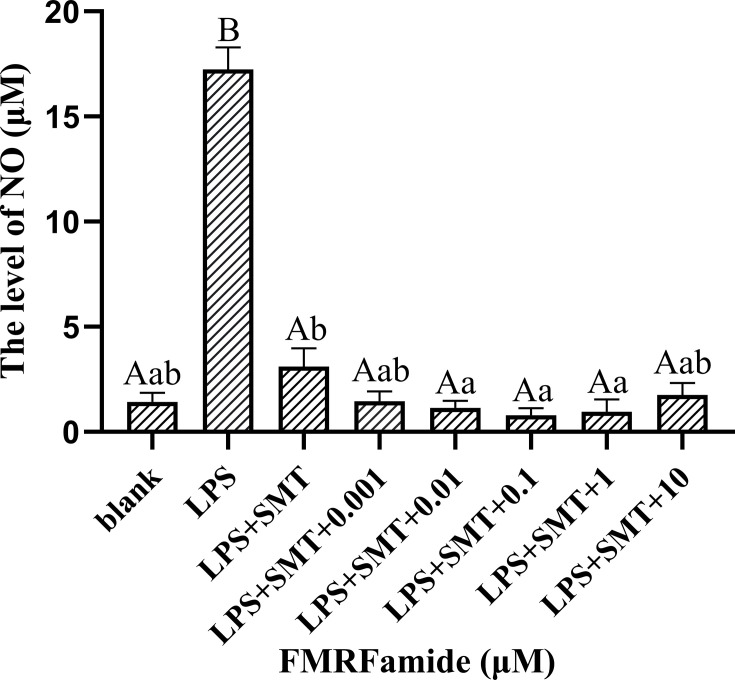
Effect of co-stimulation of SMT and FMRFamide on NO under immune stress. The graphs display the mean ± SEM (n ≥ 3), and data were analyzed by one-way ANOVA followed by Dunnett’s *post-hoc* test. The different capital letters indicate a highly significant difference (*p* < 0.01); different small letters indicate a significant difference (*p* < 0.05); the same letter indicates no significant difference (*p* > 0.05). SMT, *S*-methylisothiourea sulfate; NO, nitric oxide.

As we all know, iNOS can be induced by stress stimulation to synthesize NO. SMT selectively inhibiting iNOS was employed in this study; the co-stimulation result with FMRFamide is shown in **Figure 7**. Compared to the LPS group with a high level of NO, the addition of SMT highly significantly reduced NO production, which was expected. Compared to the LPS+SMT group of significantly reduced NO production, the addition of FMRFamide furtherly reduced NO production significantly in the range of 0.01–1 μM. The data clearly illustrated that FMRFamide could inhibit NO production through NOS-dependent and other unknown pathways.

## 4 Discussion

At present, the authentic FMRFamide has been identified in Annelida (29) and Arthropoda (30) and mostly in Mollusca (31). Reports indicate that FMRFamide has multiple regulatory functions, such as heartbeat (32), reproduction (7), feeding (33), muscle contraction (8), and glucose decrease (34). In recent years, researchers found that FMRFamide is also an important immunoregulatory factor in the NEIS to sustain homeostasis during pathogen invasion or stress response (8). FMRFamide was upregulated post *Schistosoma* parasitization in *Biomphalaria* (35). *C. gigas* FMRFamide mRNA was significantly upregulated at 12 h and then dropped back to the initial level at 24 h after LPS stimulation (8). In the present study, *V. harveyi* stimulation caused upregulation of FMRFamide precursor mRNA in just hatched cuttlefish larvae, furtherly providing proof that FMRFamide indeed participates in invertebrate immune responses. FMRFamide injection caused NO reduction in the hemolymph of *C. gigas*, suggesting that FMRFamide could also regulate NO production in the immune system (8). Our aims were to demonstrate the anatomical and physical relationships of FMRFamide with NO production under stress in cephalopod.

To visualize the possible effect of FMRFamide on NO production, double-labeling ISH was firstly applied and observed that there was an anatomical overlap of FMRFamide precursor and NOS in the CNS of *S. japonica*, the data hint that the innervation of NO production by FMRFamide containing neurons is also likely to exist in cephalopod. *H. lucorum* NOS-positive cell bodies and initial axon segments of neurons in the procerebra, medial mesocerebra, pedal, and visceral ganglia also have FMRFamide-immunolabeled fibers (12). It is speculated that the colocalization of FMRFamide with NOS might commonly exist in mollusk. These histological observations suggest FMRFamide is likely to have direct regulatory effects on NO production in multiple physiological processes.

Next, RNAi was carried out to knock down FMRFamide precursor mRNA, and the NOS expression level was highly significantly upregulated at 72 h postinfection when the FMRFamide precursor was knocked down effectively in hemocytes. Namely, FMRFamide could indeed innervate NOS expression and might furtherly control NO production. FMRFamide administration of *H. lucorum* central ganglia induced significant elevation of NO production, whereas this was dramatically inhibited by NOS inhibitor NOARG (13). Therefore, FMRFamide has direct control effects on NO production not only in the nervous system but also in the immune system.

NO generation is a feature of genuine immune-system cells and other cells involved in immune reactions; stress stimulus could evoke NO production in immune cells. NO in physiological or water conditions could react with oxygen and H_2_O to form nitrate and nitrite, both nitrate and nitrite can form light-red azo compounds, and NO content can be calculated with the photocolorimetric method (16). The NO level in *O. vulgaris* hemocytes decreased with the increase of *Aggregata octopiana* infection (36). The NO concentration and NOS activity in the hemolymph of *C. farreri* increased significantly after LPS stimulation (10). FMRFamide could enhance NO release in the nervous system of *H. lucorum* (11) and inhibit *C. gigas* hemocyte NO production (8); these reports suggest that FMRFamide has myriad regulation roles on NO in the NEIS. In the related studies, inflammatory RAW 264.7 cells were an appropriate model to assay NO production *in vitro*. From our results, the addition of FMRFamide significantly cut down NO production induced by LPS in RAW 264.7 cells; the effect was significant at low concentrations in dose- and time-dependent modes, while the effect attenuated at relatively high concentration. The inhibitory effect of FMRFamide on NO *in vitro* was consistent with *in vivo* FMRFamide injection of *C. gigas*, causing a continuous downregulation of NO content in hemolymph; the suppression effect of FMRFamide on NO production might mainly be due to the inhibition of NOS activity (37). The reduction of NO might contribute to hemocyte apoptosis (8, 10). Among the behaviors of immunocytes, apoptosis occupies a prime role in the adequate clearance of infected, damaged, and exhausted cells, especially when the hosts suffer from infection and dissemination (38). Secondly, the studies of symbiotic relationships showed that NO and NOS appeared to be attenuated in the symbiotic light organ of *Euprymna scolopes* as early as 6 h after *Vibrio fischeri* colonization to limit the aggregation of *V. fischeri* population (39, 40). NO has been considered to alter the redox status of hemolymph and influence hemocyte apoptosis in mollusks (8, 41, 42). FMRFamide-like peptide (FLP) family member neuropeptide FF can also inhibit NO release to exert contractile activity in the mouse distal colon (43).

nNOS and endothelial NOS (eNOS) produce constitutive NO at low levels (nM range), while iNOS can be induced by stress stimulations to synthesize NO at a higher level (μM range). Only one form of NOS has been identified in invertebrates (10, 44). iNOS inhibitor SMT could completely inhibit NO evoke induced by LPS in RAW 264.7 cells in our study; above all, the inhibitory effect of adding FMRFamide on NO was aggravated, illustrating that the inhibitory effect of FMRFamide on NO production during immune response not only could be reached by NOS dependent pathway but also have a similar effect in a NOS-independent manner. In brief, the data demonstrate that FMRFamide is an endogenous inhibitor with a stronger inhibition effect on NO production than chemical SMT in NEIS to sustain homeostasis.

Once infected, the organism can quickly initiate an immune response to defend against infection. One strategy is pathogen-specific, and NO is one of the key oxidative molecules that could elevate the oxidative level and cytotoxicity to destruct intruding pathogens. The other is to prevent pathogens from colonization. NO attenuation can prevent colonization from pathogen bacteria (45, 46). Therefore, the inhibitory effects of FMRFamide on NO production during the immune process can not only supervise the immune response at an appropriate level to protect autogenous normal cells from being damaged, but also help to prevent the colonization of infected pathogens to sustain homeostasis. Therefore, the inhibitory role of FMRFamide under infection needs to be initiated at the late stage of the immune response. Our above-described results including upregulation of FMRFamide precursor at 72 h postinfection by *V. harveyi* and inhibition of NO production under immune stimuli all confirmed this speculation.

## Data Availability Statement

The raw data supporting the conclusions of this article will be made available by the authors, without undue reservation.

## Ethics Statement

The animal study was reviewed and approved by the ethics committee of Zhejiang Ocean University and the Academy of Experimental Animal Center of Zhejiang Ocean University.

## Author Contributions

LZ conceived and designed the study. LZ, JQ, and HC performed the investigation and analyzed the data. LZ led the writing—original draft. CC and LZ reviewed, revised, and edited the paper. CC contributed the reagents and materials. All authors contributed critically to the article and approved the submitted version.

## Funding

This work was supported by the National Natural Science Foundation of China (NSFC; No. 31872547) and the Natural Science Foundation of Zhejiang Province, China (LY20C190007).

## Conflict of Interest

The authors declare that the research was conducted in the absence of any commercial or financial relationships that could be construed as a potential conflict of interest.

## Publisher’s Note

All claims expressed in this article are solely those of the authors and do not necessarily represent those of their affiliated organizations, or those of the publisher, the editors and the reviewers. Any product that may be evaluated in this article, or claim that may be made by its manufacturer, is not guaranteed or endorsed by the publisher.
